# A Neurological Curtain Unmasking Rheumatic Carditis in Early Adolescents: Two Illustrative Cases From a Tertiary Care Center in Maharashtra, India

**DOI:** 10.7759/cureus.90746

**Published:** 2025-08-22

**Authors:** Smita V Mohod, Priyanka M Chandankhede, Khushboo Agarwal, Gopal Agrawal, Pravin Salame

**Affiliations:** 1 Department of Microbiology, Indira Gandhi Government Medical College and Hospital, Nagpur, IND; 2 Department of Medicine, Indira Gandhi Government Medical College and Hospital, Nagpur, IND

**Keywords:** acute rheumatic fever, anti-streptolysin o, group a streptococcus, rheumatic carditis, sydenham’s chorea

## Abstract

Group A β-hemolytic streptococcal pharyngitis, if inadequately treated, can trigger an autoimmune inflammatory response known as acute rheumatic fever (ARF). While ARF classically presents with migratory arthritis, carditis, and other Jones criteria, it may also manifest as Sydenham’s chorea, a delayed neurological complication. In some patients, chorea can occur as the sole clinical feature, making diagnosis challenging. Importantly, these individuals may harbor subclinical carditis, valvular inflammation, and dysfunction detectable only by echocardiography, which, if missed, can progress to chronic rheumatic heart disease (RHD), a major cause of morbidity and mortality in resource-limited settings. We report early adolescent cases presenting with Sydenham chorea as the initial and only clinical sign of ARF. Both children, enrolled in primary school and belonging to lower socioeconomic families, showed no history of arthritis or obvious signs of carditis. Although ECG findings were non-specific, 2D echocardiography revealed subclinical carditis in both cases. Laboratory results showed raised erythrocyte sedimentation rate (ESR) and high anti-streptolysin O (ASO) titers, indicating inflammatory and possible cardiac involvement. Nutritional insufficiencies were also observed, adding to the children's vulnerability. Early treatment with benzathine penicillin and symptomatic management of chorea were initiated, and families were educated about the need for long-term secondary prophylaxis. However, barriers such as the cost and availability of injections at distant healthcare centers remain major concerns. Sydenham chorea can present as an isolated sign of ARF. These cases highlight the importance of early echocardiographic evaluation and the need to strengthen primary healthcare services to ensure timely diagnosis and continuous secondary prevention, particularly in resource-limited settings.

## Introduction

Sydenham chorea, also known as St. Vitus dance, is a neurological complication of Group A β-haemolytic streptococcal infections. It is caused by an autoimmune response in which antibodies are generated against the group A carbohydrate of *Streptococcus* spp. cross-react with neuronal tissue in the basal ganglia [1]. Acute rheumatic fever (ARF) is an abnormal immunologic response to Group A *Streptococcus* (GAS) infections [2]. Sydenham chorea can occur in up to 40% of ARF cases, and although it is a less common presentation compared to polyarthritis or carditis, it remains a hallmark diagnostic feature of ARF and is a manifestation of rheumatic fever that occurs in up to 40% of patients with the disease [3].

Studies have shown an association of ARF with low socioeconomic status, overpopulation, and rural areas with limited access to healthcare [4]. After multiple episodes of rheumatic fever, progressive fibrosis of the heart valves can occur, which may lead to subclinical carditis. If this valvular heart disease remains untreated, it may progress to rheumatic heart disease (RHD), which can be fatal [5]. Globally, the incidence of RHD ranges from eight to 51 cases per 100,000 children and young adults [6].

 This series presents two pediatric male patients from a tertiary care hospital in Central India, both presenting predominantly with chorea. Doppler echocardiographic studies have shown that subclinical carditis is frequently present even when chorea is the only clinical sign. Our cases are notable for involving early adolescent males with chorea as the sole presenting feature, yet with echo-confirmed carditis ultimately diagnosed as ARF.

## Case presentation

Case 1

Clinical History and Presentation

A 13-year-old male was admitted to the Department of Medicine at a tertiary care teaching hospital in Nagpur, Maharashtra, India, with complaints of generalized weakness and abnormal involuntary movements affecting all four limbs. These symptoms had been progressively worsening over the past eight days prior to admission. The involuntary movements were characterized as choreiform, non-rhythmic, abrupt, purposeless jerky motions that significantly interfered with the patient’s ability to carry out routine activities such as writing, eating, and walking. The patient reported a preceding history of febrile illness accompanied by a sore throat approximately two weeks before the onset of neurological symptoms. There was no prior personal or family history suggestive of any neurological or psychiatric disorder. The child belongs to a socioeconomically disadvantaged background; both parents are employed as daily wage laborers at a local construction site, with limited access to healthcare resources.

On clinical examination, the patient's vital parameters were within normal limits: blood pressure was 110/70 mmHg, pulse rate 86 beats per minute, and oxygen saturation (SpO₂) measured 98% on room air. General physical examination did not reveal any signs of systemic illness. Neurological evaluation demonstrated classical features of Sydenham’s chorea, including hypotonia, emotional lability, and motor impersistence. The choreiform movements were continuous and generalized, affecting both proximal and distal muscle groups. Fundoscopic examination showed no evidence of papilledema or raised intracranial pressure. Initial cardiovascular examination did not reveal any murmurs or signs of carditis. Routine laboratory investigations, including total leukocyte count, platelet count, renal function tests, and ionized calcium, were within normal limits (Table 1). MRI of the brain and spine was unremarkable. Electrocardiogram revealed no abnormalities. 2D Doppler Echocardiography revealed mild mitral regurgitation and mild aortic regurgitation with mild pericardial effusion without tricuspid/pulmonary involvement, while the left ventricle showed 60% ejection fraction. Throat swab and blood cultures were negative for GAS.

**Table 1:** Laboratory investigations of case 1 SGOT: serum glutamic oxaloacetic transaminase, SGPT: serum glutamate pyruvate transaminase, AST: aspartate transaminase, ALT: alanine aminotransferase, ESR: erythrocyte sedimentation rate, CRP: C-reactive protein

Sr. No.	Parameters	Result	Normal reference range
1	Hemoglobin	8.3 g/dL (Low)	12–16 (F) / 14–18 (M)
2	SGOT (AST)	69 IU/L (Elevated)	0–35 U/L
3	SGPT (ALT)	72 IU/L (Elevated)	4–36 U/L
4	Albumin	2.4 g/dL (Low)	3.5–5.5 g/dL
5	Globulin	4.0 g/dL (Low); A/G reversal	2.0–3.5 g/dL
6	Serum chloride (Cl⁻)	93.7 mmol/L (Low)	96–106 mmol/L
7	ESR	70 mm/ hr (Elevated)	0–20 mm/hr
8	CRP	47.4mg/L (Elevated)	0.08–3.1 mg/L
9	ASO titer (By Latex agglutination test with kit Brand name - BIOGENIX & lot no. ASOL0522-50)	600 IU/mL (Elevated)	<200 IU/mL

Diagnosis

The diagnosis of ARF was established based on the Revised Jones Criteria, incorporating both clinical features and supportive laboratory investigations. The patient fulfilled two major criteria: carditis (evident on 2D Doppler echocardiographic evaluation) and Sydenham’s chorea, a characteristic neurologic manifestation of ARF. In addition, the minor criteria included elevated acute-phase reactants such as ESR and C-reactive protein (CRP), along with documented fever. The constellation of these findings confirmed the diagnosis of ARF with carditis and Sydenham’s chorea.

Management

The patient was initiated on a comprehensive treatment regimen targeting the underlying streptococcal infection, inflammation, and neurologic manifestations, alongside secondary prophylaxis and supportive care. 

Antibiotic Therapy

The initial antimicrobial coverage included intravenous ceftriaxone to ensure effective eradication of any residual GAS. This was subsequently transitioned to oral penicillin V, administered at a dose of 250 mg three times daily for three days.

Secondary Prophylaxis

To prevent recurrence of ARF, the patient was started on intramuscular benzathine penicillin G, at a dose of 1.2 million units every four weeks, in accordance with standard prophylactic guidelines.

Anti-inflammatory Therapy

For the management of carditis and systemic inflammation, the patient was prescribed tablet aspirin at a dose of 150 mg three times daily, continued for two to three weeks. Concurrently, tablet prednisolone (Omnacortil) 20 mg once daily was initiated, with a gradual taper to 5 mg every alternate day over two weeks, to mitigate inflammation while minimizing steroid-related adverse effects.

Neurologic Management

To control choreiform movements, the patient received a combination of levetiracetam and clonazepam, both known for their efficacy in managing Sydenham’s chorea. In addition, tetrabenazine 25 mg at bedtime was prescribed to further alleviate involuntary movements. Maintenance low-dose aspirin (75 mg once daily) was continued as part of supportive care.

Specialty Referral and Follow-up

The patient was referred to the Department of Cardiology at Super Specialty Hospital (SSH), Nagpur, for specialized evaluation and continued management of rheumatic carditis. A follow-up visit was scheduled 15 days post-discharge to monitor clinical progress.

Nutritional Counseling and Monitoring

The patient received appropriate nutritional counseling aimed at supporting overall recovery. Plans for regular cardiac monitoring with serial echocardiography were planned as part of the long-term follow-up protocol.

Case 2

Clinical History and Presentation

**Figure 1:**
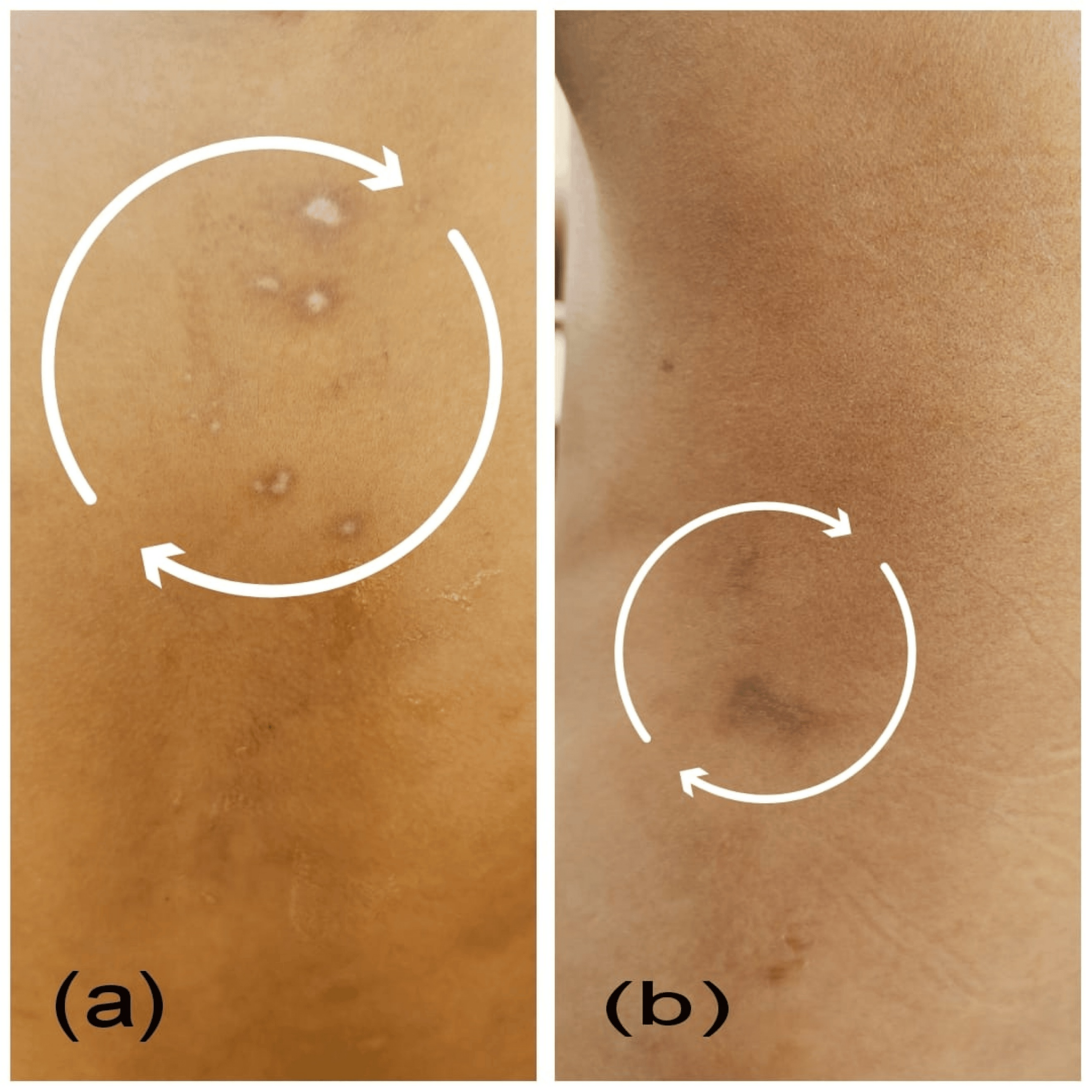
Case 2 presenting healed lesions of erythema marginatum on the chest (a) and side back (b)

A 10-year-old male was admitted to the Department of Pediatrics at a tertiary care teaching hospital in Nagpur, Maharashtra, India, with complaints of sudden-onset involuntary movements predominantly affecting the upper and lower limbs and facial muscles, along with the presence of a few skin rashes on the chest and side back (Figure 1a, 1b).

These involuntary movements were noted to intensify with voluntary actions and subsided completely during sleep (Video 1). There was no accompanying history of fever and sore throat. The child’s developmental history revealed delayed attainment of milestones, and a past history of head injury was also noted.

**Video 1:** Case 2 presenting Sydenham's chorea Patient exhibits involuntary,irregular,jerky movements in upper and lower limbs indicating Sydenham's chorea

On clinical examination, his vital signs were within normal limits, with a pulse rate of 76 beats per minute, a respiratory rate of 24 breaths per minute, and an oxygen saturation (SpO₂) of 98% on room air. Anthropometric measurements recorded a height of 129 cm and a weight of 21.1 kg, corresponding to a body mass index (BMI) of 12.7 kg/m², indicating undernutrition. Neurological assessment demonstrated classical features of Sydenham’s chorea, including choreiform movements, hypotonia, and emotional lability. Other systemic examinations were unremarkable. Ophthalmological evaluations, including fundus examination and slit-lamp assessment, were within normal limits.

Nutritional assessment revealed that the child’s average daily energy intake was approximately 1,195 kilocalories, which is below the age-appropriate requirement of 1,450 kilocalories per day. In addition, his protein intake was found to be suboptimal.

**Figure 2:**
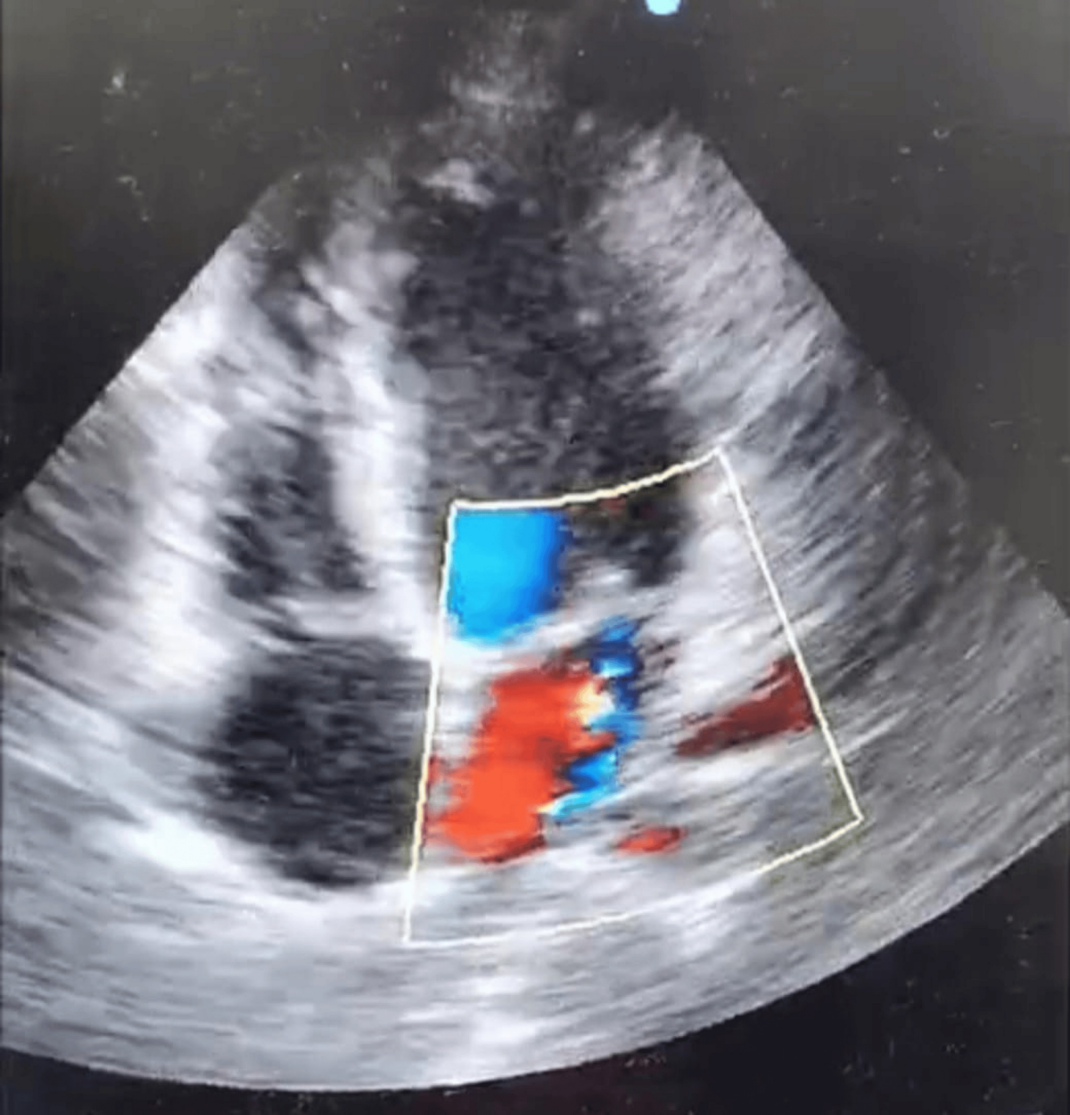
In the parasternal long axis (PLAX) echocardiographic view, the color Doppler demonstrates mitral regurgitation indicating rheumatic impression of carditis. The PLAX color Doppler view shows a significant turbulent (mosaic) flow jet across the mitral valve region. This is classic for mitral regurgitation, indicating blood is leaking from the left ventricle back into the left atrium during systole.

Routine laboratory investigations, including hemoglobin, total leukocyte count, platelet count, SGOT, sodium, potassium, and CRP, were within normal limits (Table 2). MRI of the brain and spine was unremarkable. Electrocardiogram revealed no abnormalities. 2D Doppler echocardiography revealed moderate mitral regurgitation without pulmonary involvement, while the left ventricle showed 60% ejection fraction (Figure 2, Video 2). Throat swab and blood cultures were negative for GAS.

**Table 2:** Laboratory Investigations of case 2 SGPT: serum glutamic-pyruvic transaminase, ESR: erythrocyte sedimentation rate

Sr. No.	Parameters	Result	Normal reference range
1	SGPT	9 IU/L (Low)	10–40 IU/L
2	Total protein	5.5 g/dL (Low)	6.0–8.0 g/dL
3	Albumin	3.3 g/dL (Low)	3.5–5.0 g/dL
4	Urea	11 mg/dL (Low)	15–40 mg/dL
5	Creatinine	0.4 mg/dL (Low)	0.5–1.0 mg/dL
6	ESR	70 mm/ hr (elevated)	<20 mm/hr
7	ASO titer (By Latex agglutination test with kit Brand name - BIOGENIX & lot no. ASOL0522-50)	200 IU/mL (elevated)	<150–166 IU/mL

**Video 2:** 2D echo video indicating rheumatic impression of carditis Color Doppler echocardiography, apical four-chamber view, showing a prominent systolic mitral regurgitant jet (mosaic turbulence) directed into the left atrium. The jet extends more than 4 cm and occupies nearly half of the left atrial area, indicating moderate-to-moderately severe regurgitation in the context of subclinical rheumatic heart disease.

*Diagnosis* The patient was diagnosed with ARF, presenting with carditis, Sydenham’s chorea, and erythema marginatum, fulfilling the Revised Jones Criteria. The major criteria satisfied were the presence of chorea and carditis, with the latter manifesting as mitral regurgitation evident on 2D echocardiography. The minor criteria included an elevated erythrocyte sedimentation rate and a positive anti-streptolysin O titre, both supportive of a recent streptococcal infection and systemic inflammation.

Management

The patient was initiated on oral amoxycillin-clavulanate (Amoxyclav) 625 mg, given as three-fourths of a tablet twice daily for eight days to ensure eradication of any residual streptococcal infection. For long-term secondary prophylaxis, intramuscular benzathine penicillin 6 lakh units was administered, to be repeated monthly.

To address the inflammatory component, aspirin 150 mg was started and gradually titrated up to two tablets three times daily (TID) to control joint and cardiac inflammation. In view of neuropsychiatric symptoms suggestive of chorea, the patient was given haloperidol 0.5 mg twice daily (BD) for symptomatic control. Nutritional support was provided in the form of syrup multivitamins and calcium supplements.

On discharge, the treatment plan included continuation of tablet haloperidol 0.5 mg once daily and aspirin 150 mg TID for five days, followed by a tapering schedule based on clinical assessment. Monthly intramuscular injections of benzathine penicillin were advised for secondary prophylaxis. Follow-up in the outpatient department (OPD) was planned with a repeat 2D echocardiography after two months to reassess cardiac function. Supportive therapy with syrup Tonoflex and vitamin C supplementation was also prescribed at discharge.

These cases illustrate the varied clinical spectrum of ARF, with Sydenham’s chorea as the primary manifestation in both. Despite the absence of classical features like polyarthritis or subcutaneous nodules, these cases show how chorea may precede or occur in isolation from other classic features of ARF, emphasizing the importance of high clinical suspicion in the case of ARF.

## Discussion

Rheumatic heart disease (RHD) remains the only entirely preventable cardiovascular condition that continues to cause considerable morbidity and mortality, particularly in low- and middle-income countries (LMICs) [7]. In this case series, both adolescent patients belonged to low socioeconomic households as per the Kuppuswamy classification [8], with parents working as laborers. Such socioeconomic circumstances limit healthcare access and lead to the neglect of minor infections, especially streptococcal pharyngitis. These observations are consistent with the findings of Jacob et al. (2022), who documented a similar association between low socioeconomic status and the progression of streptococcal infections to ARF and subsequent RHD [9]. Despite a global decline in ARF and RHD incidence, these diseases remain endemic in regions characterized by overcrowding, poor sanitation, and inadequate healthcare infrastructure [10]. In India, the prevalence of RHD among school-aged children ranges from 0.6 to 4.5 per 1,000 [11], which includes the age group of our cases, one in the sixth standard and the other in the fourth standard, when children are more susceptible to streptococcal infections due to environmental exposures and limited hygiene awareness.

Both patients in this study presented with Sydenham chorea as the sole clinical manifestation of ARF, a finding in line with reports by Thapa S et al. (2024) [12] and Ajith et al. (2025)[13]. This presentation reflects the diverse clinical spectrum of ARF, where chorea may occur in isolation without fever, arthritis, or overt clinical carditis. Despite the absence of classical features, both children fulfilled the Revised Jones Criteria for ARF diagnosis [14]. Subclinical carditis, which was not evident on ECG, was detected through 2D Doppler echocardiography, reinforcing the critical role of echocardiographic screening in all suspected ARF cases, particularly in endemic and resource-limited settings. Studies from high-prevalence regions indicate that subclinical carditis is present in 20-50% of chorea-only ARF cases, underscoring the importance of routine echocardiography [15].

The pathophysiological basis for this association lies in autoimmune-mediated basal ganglia dysfunction triggered by molecular mimicry between GAS antigens and neuronal tissue. The same cross-reactive antibodies implicated in Sydenham chorea may also target cardiac myosin and valvular endothelium, producing the inflammatory changes characteristic of rheumatic carditis [6]. This shared immunopathogenesis explains the coexistence of subclinical carditis with neurological symptoms, even in the absence of clinical cardiac findings. In the present cases, elevated SGOT may have been an indirect biochemical indicator of myocardial involvement.

Laboratory findings in both children revealed elevated ESR and ASO titres, consistent with recent GAS infection and active inflammation. Minor nutritional deficiencies and borderline biochemical parameters reflected a vulnerable baseline health status, increasing susceptibility to ARF and its sequelae. While microbiological confirmation remains the gold standard for GAS diagnosis, limited access and cost constraints in many LMICs hinder its routine application, making reliance on clinical algorithms common in resource-limited settings [16].

From a public health perspective, the socioeconomic background of these children mirrors patterns in other LMIC settings, where overcrowding, poor sanitation, and restricted healthcare access sustain the cycle of GAS transmission and late ARF diagnosis [9.10]. The WHO guideline on the prevention and diagnosis of rheumatic fever and RHD emphasizes a comprehensive three-pronged strategy: primary prevention through timely identification and treatment of suspected group A (β-hemolytic) *Streptococcus *(GAS) pharyngitis and skin infections; secondary prevention via long-term antibiotic prophylaxis, adherence-support interventions, and screening for early RHD; and acute management of rheumatic fever using anti-inflammatory drugs [17].

The prognosis for chorea-only ARF is generally favourable in terms of neurological recovery, with symptoms typically resolving within months; however, the coexistence of subclinical carditis substantially increases the risk of chronic valvular disease [15]. Early institution of secondary prophylaxis, as recommended by both the American Heart Association (AHA) and WHO, is essential to prevent recurrent GAS infections and halt the progression to clinically significant RHD [18]. For patients with carditis, even when subclinical, current guidelines recommend benzathine penicillin every three to four weeks for at least 10 years or until 21 years of age, whichever is longer, to protect cardiac function and optimize long-term outcomes.

However, in rural and underserved areas, logistical and financial challenges often compromise adherence to prophylaxis. This highlights the urgent need for making benzathine penicillin prophylaxis available and decentralizing 2D echocardiography at the primary health centre (PHC) level, which would significantly improve compliance and disease control in rural and underserved communities.

## Conclusions

This case series emphasizes the need for heightened clinical vigilance in identifying Sydenham’s chorea as an isolated yet definitive manifestation of ARF in children. Early 2D Doppler echocardiographic screening, even in the absence of overt cardiac symptoms, proves invaluable for detecting subclinical carditis in high-risk populations. These findings advocate for a proactive, multidisciplinary approach to diagnosis and long-term management, especially in socioeconomically challenged settings. Strengthening primary healthcare systems with accessible diagnostic tools, continuous medical education, and streamlined secondary prophylaxis programs is crucial for mitigating the long-term impact of RHD. Integrating these measures into routine pediatric care can significantly reduce diagnostic delays and improve health outcomes in endemic regions.
